# Porokeratosis ptychotropica mimicking a herpes simplex virus infection

**DOI:** 10.1016/j.jdcr.2025.08.007

**Published:** 2025-08-22

**Authors:** Erin R. Pomerantz, Jayci G. Rhein, Jason B. Lee, Sylvia Hsu

**Affiliations:** a Temple University Lewis Katz School of Medicine, Philadelphia, Pennsylvania; b Department of Dermatology, Temple University Hospital, Philadelphia, Pennsylvania; c Department of Dermatology and Cutaneous Biology, Thomas Jefferson University, Philadelphia, Pennsylvania

**Keywords:** porokeratosis, porokeratosis ptychotropica

## Introduction

Porokeratosis encompasses a group of skin disorders characterized by abnormal keratinization, with the defining histologic feature of cornoid lamellae, which are stacks of parakeratotic cells that overlie dyskeratotic keratinocytes. Several subtypes of porokeratosis have been identified, including disseminated superficial actinic porokeratosis, porokeratosis of Mibelli, linear porokeratosis, porokeratosis palmaris et plantaris disseminata, punctate porokeratosis, and porokeratosis ptychotropica (PP).^1^ Although risk factors for other forms of porokeratosis, such as UV exposure, immunosuppression, and genetic predisposition, have been identified, PP remains less understood. Clinically, PP typically presents as hyperkeratotic, verrucous plaques found bilaterally in the gluteal cleft and buttocks.^2^^,^^3^ These pruritic erythematous plaques and verrucous lesions can frequently resemble other perianal skin conditions, such as psoriasis, squamous cell carcinoma, verruca vulgaris, and candidiasis.^3^^,^^4^ This can make diagnosis more challenging, as it requires careful differentiation from other conditions when determining the most appropriate treatment. We report the case of a patient with an uncharacteristic presentation of PP that mimicked a herpes simplex virus infection.

## Case report

**Fig 1:**
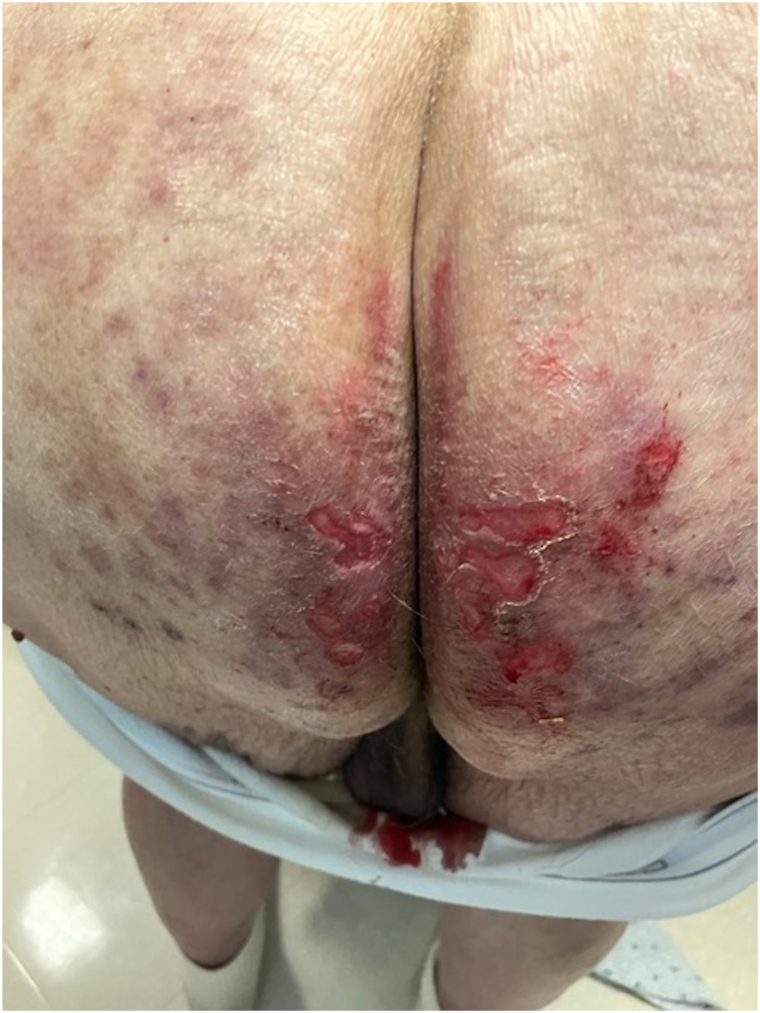
Confluent erosions with scalloped borders on both buttocks.

**Fig 2:**
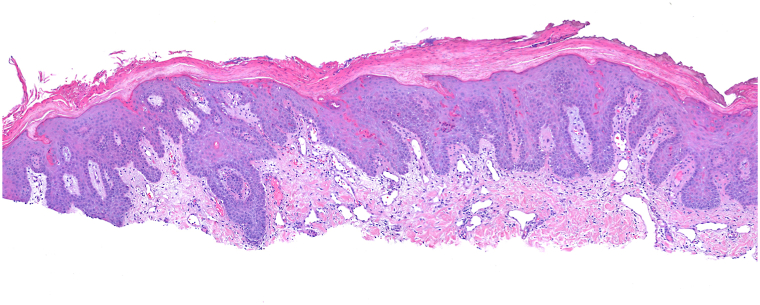
There is irregular psoriasiform hyperplasia with multiple discrete foci of dyskeratotic cells with overlying broad parakeratosis (Hematoxylin-eosin stain; original magnification: ×100).

**Fig 3:**
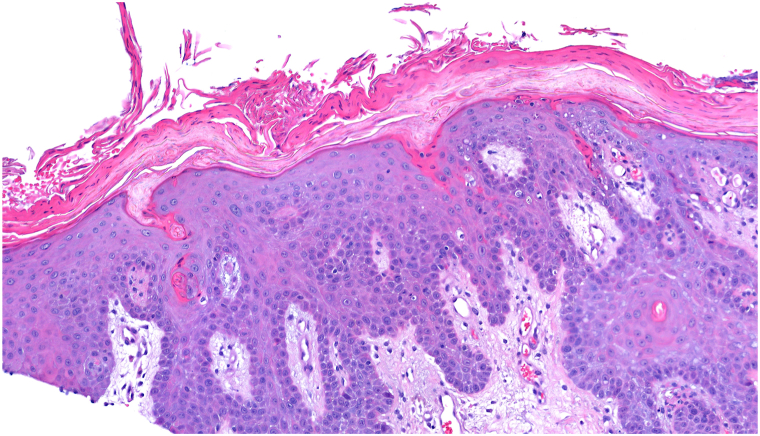
There are dyskeratotic cells in columnar arrangement with overlying parakeratosis in several foci (Hematoxylin-eosin stain; original magnification: ×400).

**Fig 4:**
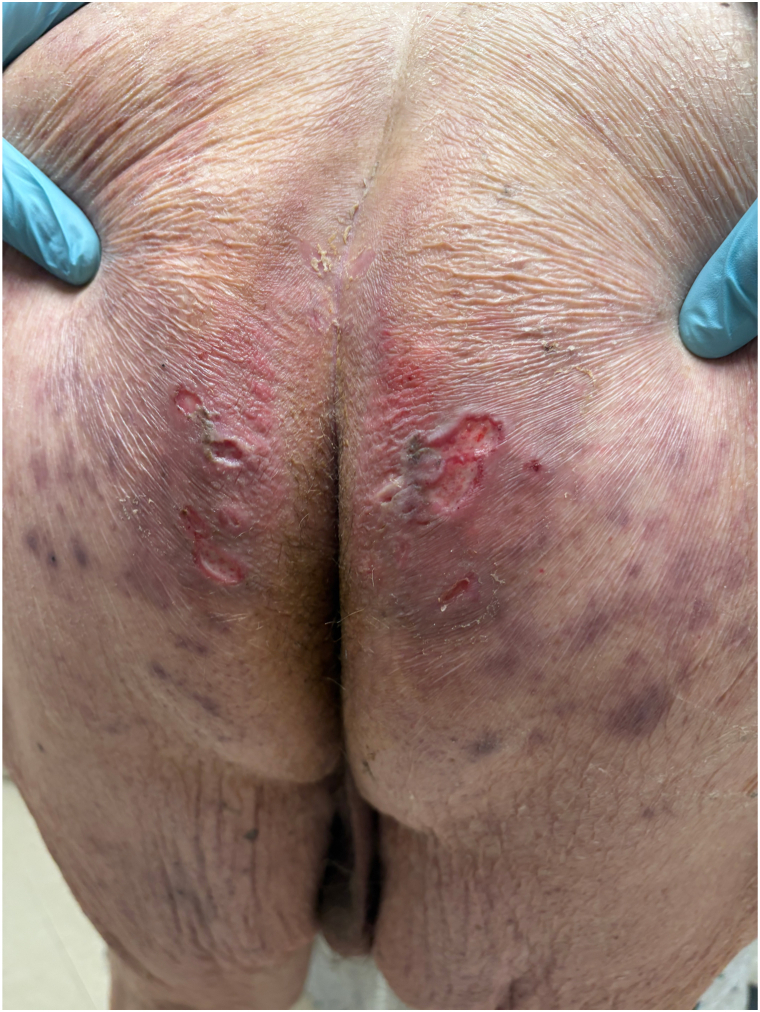
Improving erosions after 1 month of twice-daily application of 2% lovastatin.

**Fig 5:**
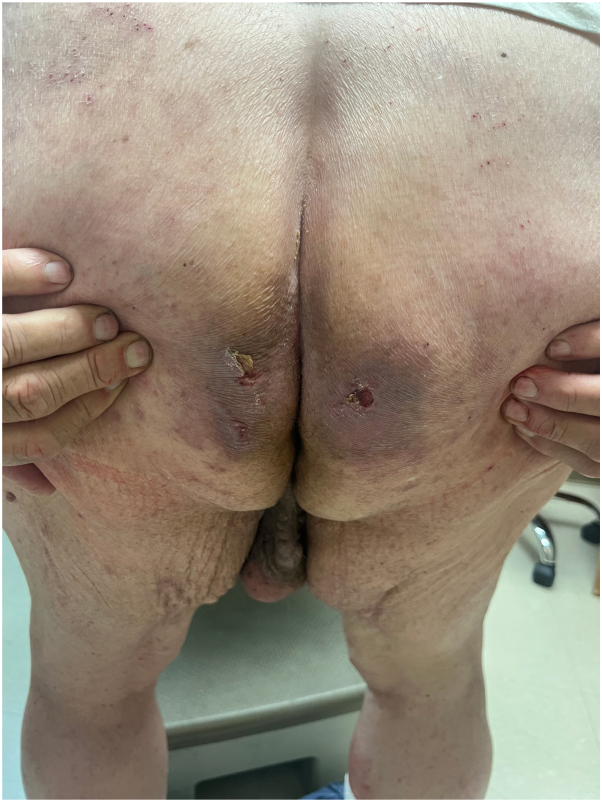
Significantly improved erosions after 3 months of topical lovastatin.

A 76-year-old male presented to the dermatology clinic with a several-week history of a painful rash on his buttocks. His past medical history was notable for stage 4 chronic kidney disease, type 2 diabetes mellitus, atrial fibrillation, and congestive heart failure. The patient was not applying any topical products to the area. On physical examination, there were confluent erosions with wavy or notched borders on both buttocks, sparing the gluteal clefts. The confluent erosions with scalloped borders raised a primary concern for herpes simplex virus infection (Fig 1). There was no purulence or induration to suggest secondary bacterial infection. A biopsy of the border of the ulcer was performed, and the patient was started on valacyclovir. The biopsy results revealed PP, and valacyclovir was discontinued. Histologic examination revealed irregular psoriasiform hyperplasia with multiple discrete foci of dyskeratotic cells and overlying broad parakeratosis (Fig 2). There were dyskeratotic cells in columnar arrangement with overlying parakeratosis in several foci (Fig 3). The patient was started on topical 2% lovastatin cream. At follow-up visits at 1 and 3 months, the erosions had significantly improved (Figs 4 and 5).

## Discussion

PP is a rare variant of porokeratosis with only 59 cases reported between 1995 and 2002, 86.4% of which occurred in males.^2^ Lucker et al^5^ first identified PP in a case in which the lesions involved body folds and differed from previously described presentations, although the characteristic histologic features of porokeratosis were still evident. Moreover, on histologic examination, PP can be distinguished by amyloid deposition and the presence of multiple cornoid lamellae in the entire lesion instead of peripherally, which is characteristic of the other subtypes of porokeratosis.^3^^,^^4^ This highlights the importance and utility of histologic evaluation in not only diagnosing porokeratosis but also distinguishing it from other subtypes.

This is important when determining treatment and ongoing surveillance because of the potential malignancy risk associated with other variants. Although the available research is limited and the findings are not fully consistent, studies have shown that porokeratosis lesions, particularly in linear porokeratosis, are prone to malignant transformation into squamous cell carcinoma and basal cell carcinoma.^2^ Studies have found that the overall incidence of malignancy is 6.8%-11.6% across all types of porokeratosis.^6^ Only 1 case of PP in the literature has been linked to squamous cell carcinoma, likely due to its rarity and potential for misdiagnosis, resulting in an overall 1.7% risk.^2^^,^^7^ The patient in that case had prior exposure to ionizing radiation for a previous malignancy, which could have contributed to the development of squamous cell carcinoma.^7^ Despite this finding, the risk of malignant transformation in PP remains poorly understood and is an important factor to consider in long-term treatment.

Kubo et al^8^ analyzed the genetic mutations associated with disseminated superficial actinic porokeratosis and linear porokeratosis and found that second-hit genetic changes, causing a cytosine-to-thymine mutation de novo or postnatally, are responsible for the development of these disorders. Notably, these genetic changes, which can also be induced by UV exposure, occur in the mevalonate pathway enzymes mevalonate decarboxylase and mevalonate kinase.^8^ Although a genetic predisposition has been observed in other variants of porokeratosis, some of which exhibit autosomal dominant inheritance, little is known about the genetic factors involved in PP.^8^ Most of the reported PP cases appear to be sporadic, with no known genetic contribution or familial linkage.^9^ Furthermore, UV exposure has not been identified as a risk factor in the development of PP.

Treatment options for PP have varied, with a range of therapies reported, although no single approach has demonstrated consistent efficacy. Topical retinoids, commonly used to address the abnormal keratinization in porokeratosis, often fail to produce a significant improvement.^2^ Other treatments that have been trialed for managing porokeratosis include surgical excision, cryotherapy, CO_2_ laser ablation, and topical agents such as corticosteroids, 5-fluorouracil, imiquimod, vitamin D derivatives, diclofenac gel, cantharidin, and tacrolimus.^1^ In addition to these treatment strategies, genetic mutations in the enzymes of the mevalonate pathway, implicated in the pathogenesis of other porokeratosis variants, offer a potential therapeutic target. Lovastatin has shown promise due to its ability to reintroduce cholesterol, the product of this pathway, and prevent the accumulation of toxic by-products.^10^ Although several treatment options exist, further research is needed to identify the most effective approach for managing PP, as success has been limited.

The atypical presentation of PP as erosions with scalloped borders, which has not been described before, emphasizes the importance of recognizing the varied clinical manifestations of this condition and the crucial role of histologic examination. Refractory ulcers, those with scaly borders and characteristic distribution on the buttocks, may serve as diagnostic clues for the clinicians. To demonstrate the histologic findings, biopsy of an ulcerated lesion should include intact epidermis. Given the rarity of PP, we hope this case offers valuable insights to clinicians in the diagnosis and management of this disorder.

## Conflicts of interest

None disclosed.
